# A Ph.D. thesis entitled “Dose audit and optimization for radiological and interventional procedures”

**Published:** 2008

**Authors:** Keith Faulkner

**Affiliations:** Quality Assurance Reference Centre, Unit 9, Kingfisher Way, Silverlink Business Park, Wallsend, Tyne and Wear NE28 9ND E-mail: keith.faulkner@nhs.net

There is increasing international concern about dose levels in diagnostic radiology. The population dose from diagnostic radiology continues to increase, as successive United Nations Scientific Committee on the Effects of Atomic Radiation surveys have demonstrated. This rise is due to an increase in both the frequency of medical radiology examinations and the number of high dose examinations such as computed tomography and interventional radiology. As a consequence, international bodies such as the International Commission on Radiological Protection (ICRP) have tried to introduce a framework for radiation protection to reduce this burden.

One of the weaknesses in the analysis of worldwide exposures undertaken by UNSCEAR is the limited data on countries such as India. Information on dose levels for people living in India is particularly important for the estimation of the worldwide population dose. The Ph.D. thesis by Dr. Livingstone is an important piece of research in this context as he has looked at patient doses for a number of examinations in an Indian setting. Dr. Livingstone has looked at dose levels for a number of different types of radiology examinations but has quite correctly concentrated on high dose procedures involving fluoroscopy and CT. The thesis contains an interesting study of technique factors and patient dosimetry for various fluoroscopy examinations such as barium studies, micturating cystourethrogram (MCUs), hysterosalpingograms, myelograms, etc. Dr. Livingstone described the results of one of the few surveys of effective doses for pediatric patients undergoing barium studies, colonograms and MCUs. This has been achieved by deducing average organ doses for these procedures.

Assessment of stochastic risks for medical exposures is complicated; because almost invariably, more than one organ is irradiated. ICRP has introduced the quantity effective dose (E) for assessing the dose to radiation workers and to the whole population. Both the radiation and tissue weighting factors are deduced from radiobiological studies. Thus, the numerical value of effective dose for a particular examination changed after ICRP revised its organ weighting factors. Thus, a major limitation of using effective dose in the assessment of medical exposures is that it may be difficult to perform trend analysis in the future if this analysis is based on effective dose. However, effective dose is commonly used to assess medical exposures.

In common with virtually all patient dose surveys, Dr. Livingstone discovered a wide range in effective dose levels for nominally the same examinations. It is not uncommon for the range in effective dose levels to a patient to vary by a factor of 10 or more. There are also detailed patient dosimetry studies for interventional procedures such as cerebral and vascular interventions and angiography. This data is particularly interesting for the evaluation of population dose.

Dr. Livingstone has also determined effective dose for a number of CT examinations, including spinal CT and those for pediatric patients. Effective doses estimated in some of the common CT examinations performed using spiral CT scanner were as high as 9.87 ± 0.16 mSv for biphasic abdomen, 6.89 ± 1.07 mSv for cholangiogram, 6.43 ± 0.73 mSv for renal angiogram, 4.65 ± 0.37 mSv for entire abdomen and 4.01 ± 0.6 mSv for routine chest; whereas for other CT examinations such as for brain (noncontrast and contrast), and for high-resolution thorax involving axial slice acquisition, the values were 0.82 ± 0.01 mSv, 0.44 ± 0.005 mSv and 0.09 ± 0.03 mSv respectively. Establishing dose levels from CT is important because in countries such as the USA, half the population dose arises from CT. Thus, its quantification is particularly relevant. The thesis shows what can be done as part of a dose audit. Dr. Livingstone has taken this a step further by investigating optimization of techniques as part of his thesis.

In summary, it is an interesting piece of work which contributes to our knowledge of population dose worldwide. Dr. Livingstone has shown how patient dose audit and optimization can be developed and implemented.

